# Isolated Kaposi Sarcoma of the Tonsil: A Case Report and Review of the Scientific Literature

**DOI:** 10.1155/2015/874548

**Published:** 2015-02-10

**Authors:** Barbara Pittore, Carlo Loris Pelagatti, Francesco Deiana, Francesco Ortu, Elena Maricosu, Sergio Cossu, Giovanni Sotgiu

**Affiliations:** ^1^Otorhinolaryngology Department, S. Francesco Hospital, ASL 3, Nuoro, Italy; ^2^Immunology Department, AOU Cagliari, Italy; ^3^Pathology Department, S. Francesco Hospital, ASL 3, Nuoro, Italy; ^4^Epidemiology and Medical Statistics Unit, Medical Education and Professional Development Unit, Department of Biomedical Sciences, University of Sassari-Research, AOU Sassari, Italy

## Abstract

Kaposi sarcoma is a tumour caused by human herpes virus 8, also known as Kaposi sarcoma-associated herpes virus. It usually affects the skin and oral mucosa; however, it can also sometimes affect the lungs, the liver, the stomach, the bowel, and lymph nodes. Several body sites may be affected simultaneously. The involvement of the tonsils is rare. We described an isolated localization of Kaposi's sarcoma of the right tonsil in a HIV-positive patient.

## 1. Introduction

HIV/AIDS represents an important clinical and public health issue worldwide. The estimated HIV/AIDS prevalence in 2012 was 35,300,000 worldwide in 2012 (WHO. Global report: UNAIDS report on the global AIDS epidemic 2013, UNAIDS/JC2502/1/E.). In case of severe immunodeficiency, the probability of occurrence of several opportunistic disorders (e.g., infections and tumours) can increase. Kaposi's sarcoma (KS) is a frequently reported neoplasia in HIV-positives; it is caused by human herpes virus 8 (HHV-8), also known as KS-associated herpes virus (KSHV) [1]. The most recurrently affected sites are the skin and mucous membrane [2, 3]; however, it can be found in other parts of the body (i.e., lungs [4], stomach [5], intestine [6], etc.). The KS of the head and neck usually involves the mucous membranes of the oral cavity [7], but it can be found in the pharynx [8, 9], larynx [10], and nose [11]. Isolated oropharyngeal location, in particular the tonsillar one, is extremely rare [12–16].

Here, we present a case of KS of the right tonsil in a HIV-positive patient, surgically treated and exposed to antiretroviral drugs.

## 2. Case Report

A 42-year-old man was admitted to the Emergency Department of the S.Francesco Hospital, Nuoro, Italy, because of a painless swelling, moderate bleeding, located in his right tonsil. He referred to its occurrence without any other relevant symptoms 1 month ahead.

**Figure 1:**
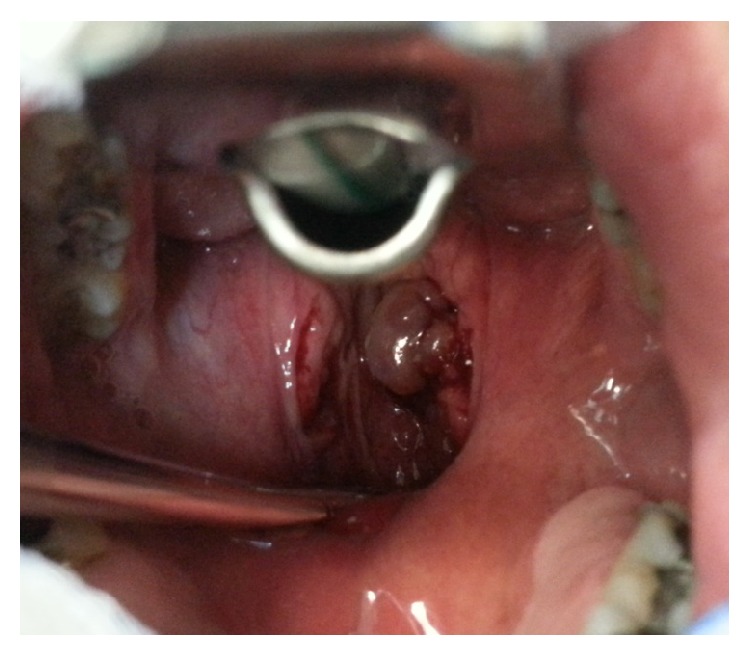
Intraoperative photo: KS involved right tonsil.

Oral examination shows a purple swelling (Figure 1) in the upper pole of his right tonsil, sized about 1.5 cm (maximum diameter). Further clinical examinations did not detect any other lesions in the head and neck district. The suspected diagnosis was a pyogenic granuloma. The patients did not reveal his known HIV positivity. He underwent a tonsillectomy with complete excision of the above mentioned tumour.

**Figure 2:**
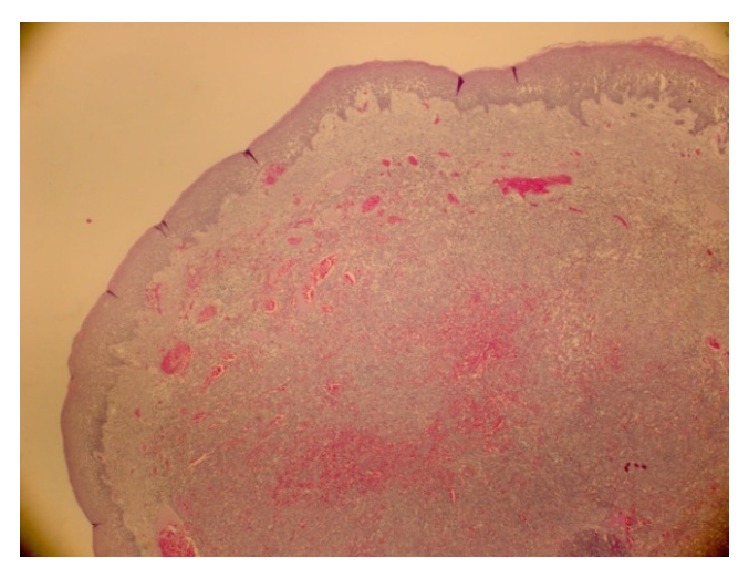
(E.E 100x). Picture that shows a nodular lesion between vascular area in the chorion under the epithelium.

**Figure 3:**
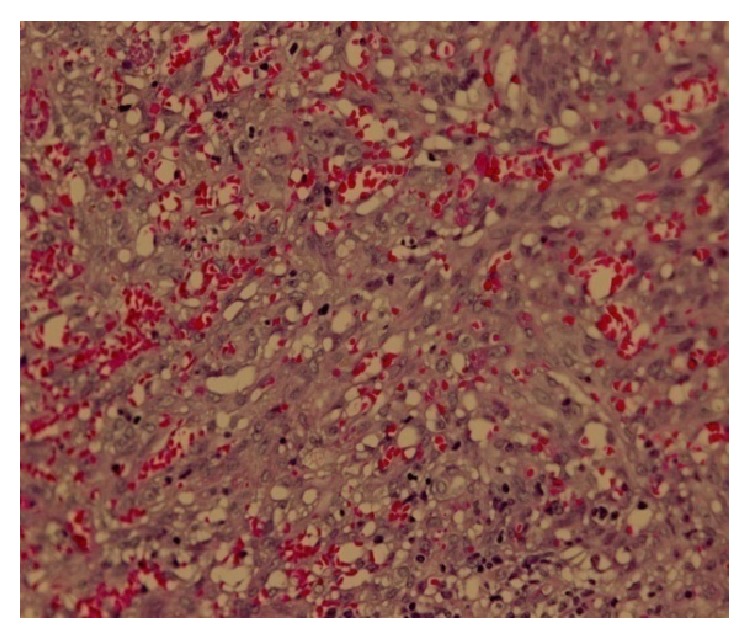
(H E 200x). KS of the tonsil demonstrates irregularly shaped vascular channels, interlacing bundles of spindle-shaped cells.

**Figure 4:**
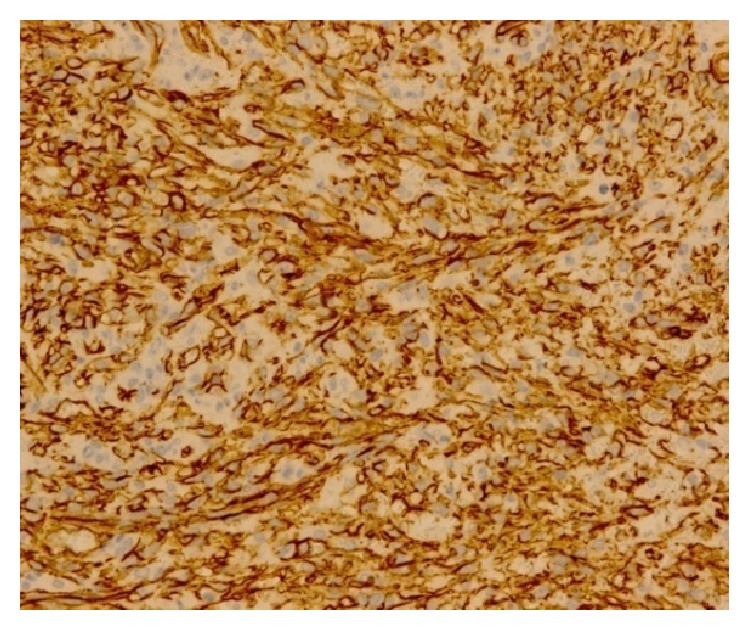
(I.I 200x). CD34 immunoreactivity is seen in the neoplastic spindle cells.

**Figure 5:**
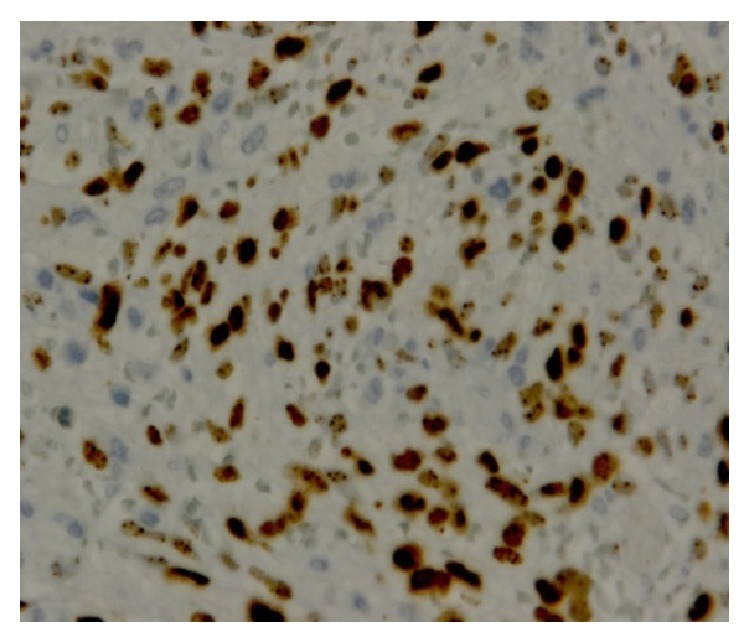
(400x). Picture that shows the nuclear human herpes 8 immunopositivity.

The histopathological analysis identified a KS (Figures 2, 3, 4, and 5). In order to better understand the etiology and the staging of the tumour a diagnostic plan was arranged: HIV-testing, endoscopy of the upper part of the digestive tract, and computer tomography scanning of the chest and abdomen.

His past medical history includes a borderline hypertension treated with a diet approach.

After surgical treatment his CD4 lymphocyte count and HIV viral load were 207 cells/*μ*L and >100,000/mL, respectively (CDC stage C2). On the basis of the TIS classification (Table 1) [17], he was classified as T0I0S0. Antiretroviral therapy (i.e., emtricitabine/tenofovir + darunavir 800 mg/Ritonavir) as well as sulfamethoxazole/trimethoprim were immediately prescribed; after five months his CD4 lymphocyte counts increased (i.e., CD4 = 283 cells/*μ*L) and the level of viral load decreased gradually (i.e., undetectable).

After 15 months since KS diagnosis the patient is alive and KS-free with a CD4 cell count of 342 cells/*μ*L.

## 3. Discussion

KS is an important opportunistic disease, which frequently occurs in HIV-positive patients characterized by a severe immunodeficiency. The KS incidence in the general population is 1 case per 100,000 inhabitants [18], whereas it is 1 per 20 inhabitants in the HIV-infected population [18]. HHV-8 is the etiological agent associated with all described KS subtypes; the prevalence of the infection is around 20–30% in Europe [18].

The AIDS-related KS lesions, affecting the skin of the upper trunk and face [2, 3], and the mucosa of the oral cavity [19], often progress rapidly to plaques and nodules. The oropharyngeal KS and, in particular, isolated KS of the tonsil are extremely rare and only a few cases were described in literature [13, 15, 16]. Raikundalia described the first case of KS located in the upper pole of the left tonsil in a female aged 38. She presented another subcutaneous lesion on her face and thighs. Oropharyngeal KS was surgically treated [12]. Chetty and Batitang described two KS cases of the tonsil in HIV-positive patients who underwent an excisional biopsy, but subsequently both patients defaulted treatment and were lost to follow-up [13]. Sikora et al. illustrated two KS cases in HIV-negative persons [14]. The first was a 49 years old man with a slow growing right tonsillar mass associated with a mobile right level II node. Fine needle aspiration of the lymphonode and the histology of the tonsil mass allow the diagnosis of a KS. After 3 years from the surgery the patient was disease-free [14]. The second patient described was a 34-year-old male showing a 2 cm sized lesion of the base of the tonsil, without any other lesion. No recurrence after 3 years from the tonsillectomy was recorded. Al-Brahim et al. [15] referred to a patient with membranous glomerulonephritis exposed to immunosuppressive therapy. After 10 months of exposure to the therapy the patient developed a tonsil KS. Saxena et al. illustrated a case of a large bilateral KS of the tonsils and adenoids in a 31-year-old man, surgically treated [16]. One of the most relevant series of KS of the head and neck was described by Yu et al. [20]. Among the 121 patients they described 10 (8.3%) showed lesions in the tonsils. Only 12 patients survived from the disease over two years for an overall survival rate of 9.92% [20].

The therapeutic choice and the following prognosis are extremely variable, depending on the stage and the KS type.

The AIDS Clinical Trials Group Oncology Committee published criteria for the evaluation of the epidemic KS [18]. The staging system includes the extent of disease (T), the immune system status (I), and presence of systemic symptoms (S) (Table 1) [17].

This classification includes two group of patients: “Good risk category (T0I0S0)” and “Poor risk category (T1I1S1).” The patient that we described was classified as T0I0S0.

KS can be treated with radiotherapy, chemotherapy, biologic agent as interferon alfa, and surgery: in HIV-positive patients it can be managed with HAART or with a combination of this therapeutic approach.

HAART seems to be indispensable in the treatment of the AIDS-related KS, alone or in combination with systemic or/and local therapy [21]. HAART can prevent the occurrence of KS or can induce regression of KS lesions. Some AIDS patients show clinical recovery and prolonged remission while continuing the therapy. Therefore, HAART should be considered first-line treatment for those patients, though in some cases they may require other concomitant treatments. This therapeutic strategy currently recommended is highly effective favouring the reduction of the HIV viral load, the increase of the CD4 T-lymphocyte count, and the decrease of the occurrence of the HIV-related tumours, such as KS. Campanini et al. in their retrospective study on 470 HIV-positive patients treated with HAART showed a reduction cancer probability (i.e., from 3% to 0%) [22]. Despite those positive effects, adverse clinical events can occur; in particular, the immune reconstitution inflammatory syndrome (IRIS), characterized by an exuberant immune-mediated inflammatory response, can increase the treatment-related morbidity [23]. It can allow the recurrence of previously treated opportunistic infections and/or neoplasm [23].

Radiotherapy is an effective palliative treatment reducing pain, bleeding, and oedema. Cutaneous KS is highly radiosensitive as described by Donato et al. in a group of 38 AIDS-associated KS lesions [24]. They show a complete and partial response of 31 lesions (83.8%) and 6 (16.2%) lesions after RT, respectively [24].

Chemotherapy (CHT) can be local (intralesional injection) or systemic (intravenous). Local CHT, based on bleomycin, cisplatin, and vinblastine, can represent an alternative therapy for limited cutaneous KS with complete or partial response rates (range: 60–92%) [25, 26]. Systemic cytotoxic agents are usually prescribed to patients not responding to HAART and/or with widespread mucocutaneous and visceral disease [27]. Several drugs, such as vincristine, vinblastine, etoposide, bleomycin, docetaxel, and paclitaxel, can be administered [18]. Treatment with paclitaxel is limited to patients with recurrent disease after a first-line CHT [18].

Interferon alfa, showing immunomodulatory, antiviral, and antiangiogenetic actions, can be used alone or in combination with HAART. It can favour a remission of the KS lesions in 20 to 60% of the exposed cases [28, 29]. The probability of a positive outcome can significantly increase in case of a therapy longer than 6 months. Its administration should not be considered in case of progressive or visceral disease.

Patients with clinical progression despite CHT and/or HAART can be currently approached with the so-called “Target Therapy” (i.e., antiangiogenetic agents such as inhibitor of vascular endothelial growth factor (VEGF) and thalidomide, metalloproteinases (MMPs), and cytokine signalling pathway inhibitors) [18]. MMPs are a family of zinc-dependent endopeptidases involved in the destruction of the extracellular matrix proteins [30], constitutively over-expressed in KS cells, and facilitate tumour invasion and metastatic localizations [18, 30]. COL-3 can inhibit the activity, activation, and production of MMPs [18, 30]. IL-12 has showed anti-tumour and antiangiogenic activity, downregulating an active G protein coupled receptor encoded by KSHV [18, 31].

Biopsy of the lesion is deemed mandatory for the histological diagnosis, but in cases of isolated cutaneous and visceral KS surgical excision is recommended in association with HAART, as showed in our review [12, 14, 16].

The patient we managed underwent tonsillectomy and HAART with complete eradication of the tumours.

## 4. Conclusion

Isolated KS of the tonsil is very rare and can simulate benign or malignant lesions, such as pyogenic granuloma, carcinomas, and sarcomas. Histological diagnosis, HIV testing and CT scans of the chest and abdomen, and an endoscopy of the upper gastrointestinal tract can be helpful for an appropriate stage of the tumours and, consecutively, to plan a better therapeutic approach.

## Figures and Tables

**Table 1:** TIS classification [17].

TIS classification	Clinical categories	
	Good category	Poor category
T	Skin, lymphonode, mucosa of the mouth	Oedema and ulceration, KS of the mouth, KS of the gastrointestinal tract, KS of the viscera

I	CD4 cells count ≥200 cells/*μ*L	CD4 cells count 200 cells/*μ*L

S	No history of opportunistic disease; no fever, loss of weight, persistent diarrhea	Disease as lymphoma, neurological disease, fever, and so forth
